# Epigenetic control of cell identities from epiblast to gastrulation

**DOI:** 10.1111/febs.70024

**Published:** 2025-02-22

**Authors:** Katrin M. Schüle, Simone Probst

**Affiliations:** ^1^ Faculty of Medicine, Institute of Experimental and Clinical Pharmacology and Toxicology University of Freiburg Germany; ^2^ Signaling Research Centers BIOSS and CIBSS University of Freiburg Germany

**Keywords:** DNA methylation, embryonic stem cells, epigenetics, gastrulation, germ layers, histone modifications, mouse embryo, nucleosome remodeling, transcription factors

## Abstract

Epigenetic modifications of chromatin are essential for the establishment of cell identities during embryogenesis. Between embryonic days 3.5–7.5 of murine development, major cell lineage decisions are made that discriminate extraembryonic and embryonic tissues, and the embryonic primary germ layers are formed, thereby laying down the basic body plan. In this review, we cover the contribution of dynamic chromatin modifications by DNA methylation, changes of chromatin accessibility, and histone modifications, that in combination with transcription factors control gene expression programs of different cell types. We highlight the differences in regulation of enhancer and promoter marks and discuss their requirement in cell lineage specification. Importantly, in many cases, lineage‐specific targeting of epigenetic modifiers is carried out by pioneer or master transcription factors, that in sum mediate the chromatin landscape and thereby control the transcription of cell‐type‐specific gene programs and thus, cell identities.

## Graphical abstract

In this review, we present and contextualize current knowledge about the roles of epigenetic modifiers during the development of mouse epiblast to gastrulation stage. The epigenetic regulation of enhancer and promoter elements by DNA methylation, histone modifications, and chromatin accessibility in concert with lineage‐specific transcription factors is crucial for correct lineage‐specific gene expression.

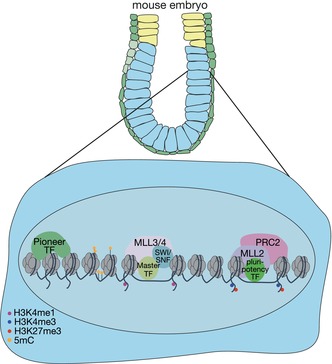

**Abbreviations**
(i)ETX: (induced) stem cell‐based embryo model generated by assembly of ESCs, TSCs, and extraembryonic endoderm stem cells (XENs)
(sc)ATAC: (single‐cell) assay for transposase‐accessible chromatin
3D: three‐dimensional
5caC: 5‐carboxylcytosine
5fC: 5‐formylcytosine
5hmC: 5‐hydroxymethylcytosine
5mC: methylcytosine
ac: acetylation
ActA: Activin A
ASCL1: achaete‐scute family bHLH transcription factor 1
ATP: adenosine triphosphate
ATPase: adenosine triphosphatase
AVE: anterior visceral endoderm
BAF: BRG1/BRM‐associated factor
BMP: bone morphogenetic proteins
BRD4: bromodomain 4
BRG1: brahma‐related‐gene 1
BRM: brahma
Cas9: clustered regularly interspaced short palindromic repeats (CRISPR)‐associated protein 9
CBP: CREB‐binding protein
CHD: chromodomain helicase DNA‐binding
ChIP: chromatin immunoprecipitation
CpG: cytosine‐phosphate‐guanine dinucleotide
CRE: cis‐regulatory element
dCas9: catalytically dead Cas9
DE: definitive endoderm
DNA: deoxyribonucleic acid
DNMT: DNA methyltransferase
DVE: distal visceral endoderm
E: embryonic day
EB: embryoid body
EED: embryonic ectoderm development
EiTiX: stem cell‐based embryo model generated by assembly of ESCs, induced TSCs, and induced XENs
ELF5: E74‐like ETS transcription factor 5
Epi‐LCs: epiblast‐like cells
EpiSCs: epiblast stem cells
esBAF: ESC‐specific BAF complex
ESCs: embryonic stem cells
ETS: stem cell‐based embryo model generated by assembly of ESCs and trophoblast stem cells (TSCs)
ExE: extraembryonic ectoderm
ExEn: extraembryonic endoderm
ExM: extraembryonic mesoderm
EZH1/2: enhancer of zeste homolog 1/2
FGF: fibroblast growth factor
FOXA1/2: forkhead box A1/2
GATA6: GATA binding protein 6
GRN: gene regulatory network
GSK3β: glycogen synthase kinase‐3 beta
H3K27: histone 3 lysine 27
H2AK119: histone 2A lysine 119
H3K4: histone 3 lysine 4
H3K9: histone 3 lysine 9
HAT: histone acetyltransferase
HDAC1/2: histone deacetylase 1/2
HMT: histone methyltransferase
ICM: inner cell mass
INO80: INOsitol‐requiring mutant 80
ISWI: imitation switch
KDM: histone lysine demethylase
KMT2A‐D: histone lysine N‐methyltransferase 2A‐D
KO: knockout
LEFTY1/2: left–right determination factor 1/2
LHX1: LIM homeobox 1
LIF: leukemia inhibitory factor
LMR: lowly methylated region
MBD2/3: methyl‐CpG‐binding domain 2/3
ME: mesoderm and definitive endoderm
me1: mono‐methylation
me3: tri‐methylation
MEK: mitogen‐activated protein kinase
MESP1: mesodermal posterior 1
MIXL1: mixed paired‐like homeobox 1
MLL1‐4: mixed lineage leukemia 1–4
NE: neuroectoderm
NGS: next‐generation sequencing
NuRD: Nucleosome remodeling and deacetylase
OCT4/6: octamer‐binding transcription factor 4/6
PcG: polycomb group
PCGF1‐6: polycomb group RING finger 1–6
PE: primitive endoderm
PGCs: primordial germ cells
POU3F1: POU domain, class 3, transcription factor 1
POU5F1: POU domain, class 5, transcription factor 1
PRC1/2: polycomb repressive complex 1/2
PS: primitive streak
RA: retinoic acid
RBBP4/7: retinoblastoma‐binding protein 4/7
REST: RE1‐silencing transcription factor
RING1A/B: RING finger protein 1A/B
RNA pol II: RNA polymerase II
RNA: ribonucleic acid
RNF2: RING finger protein 2
scRNA: single‐cell RNA
seq: sequencing
SET1: Su(var)3–9, enhancer of zeste, and trithorax 1
SETD1A: SET domain containing 1A
SFRP4: secreted frizzled‐related protein
SMARCA2/4: SWI/SNF related, matrix associated, actin dependent regulator of chromatin, subfamily a, member 2/4
SOX2: *sex‐determining region Y(SRY)‐box 2*
STAT3: signal transducer and *activator of transcription 3*
SUZ12: suppressor of zeste 12 protein homolog
SWI/SNF: switch/sucrose nonfermentable
TE: trophectoderm
TET: ten‐eleven translocation (methylcytosine dioxygenases)
TF: transcription factor
TFIID: transcription factor II D
TKO: triple knockout
TSS: transcription start site
VE: visceral endoderm
WGBS: whole‐genome bisulfite sequencing
WNT: wingless‐related integration site
ZIC2/3: zinc finger protein of cerebellum 2/3

## Introduction

The regulation of cell‐type and cell‐state‐specific transcriptional programs during mouse embryogenesis from the blastocyst to the gastrulation stage embryo is coordinated by the interplay of signaling pathways, transcription factors (TFs), and epigenetic modifiers (reviewed by [1, 2, 3, 4]). These cooperatively regulate gene expression at cis‐regulatory elements (CREs), such as promoters and gene‐specific enhancers. Chromatin at these CREs is characterized by specific patterns of DNA and histone modifications and different levels of chromatin accessibility. These epigenetic CRE characteristics impact the interaction of many transcriptional regulators with their genomic DNA consensus sites (reviewed by [5, 6]). Different patterns of chromatin marks at CREs are associated with transcriptional activity of the corresponding genes and thus are characteristics for active or repressive chromatin (reviewed by [7]). However, to date the mechanistic understanding of how the dynamics of different chromatin states contribute to the regulation of lineage‐specific gene expression, and if these are indeed instructive for transcriptional regulation or rather represent a consequence following transcription control at gene‐specific loci, remains largely unclear.

**Fig. 1:**
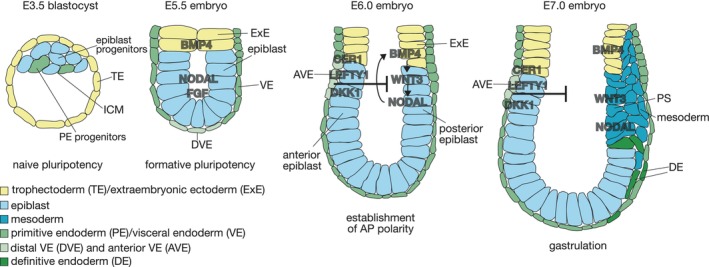
Mouse development from blastocyst to gastrulation. At E3.5 the blastocyst consists of two lineages, the trophectoderm (TE) and the inner cell mass (ICM). The TE (yellow) will differentiate into the extraembryonic ectoderm (ExE) (yellow) and will give rise to the embryonic part of the placenta. The ICM contains the epiblast progenitors (light blue) which will later give rise to the embryo proper and the primitive endoderm (PE) progenitors (green). Between E4.5 (not shown) and E5.5 the PE will differentiate into the visceral endoderm (VE) (green). By E4.5 (not shown) the embryo implants into the uterus. In the postimplantation embryo, the epiblast (light blue) will form a single epithelial layer that proliferates and adopts the typical cup‐shaped morphology of the mouse embryo and is covered by the VE. At E5.5 the epiblast is in a formative pluripotent state, identified by downregulation of naïve pluripotency markers. Core pluripotency transcription factors like *Nanog*, *Pou5f1*, and *Sox2* remain expressed (not depicted in the figure). Furthermore, this state is characterized by NODAL and FGF signaling in the epiblast and BMP4 signaling in the ExE and the absence of WNT signaling. NODAL signals induce the formation of the distal VE (DVE) (light green), which expresses inhibitors of NODAL and WNT signaling (CER1, LEFTY1, and DKK1). By E6.0, the DVE has moved to the future anterior side of the embryo to form the anterior VE (AVE) (light green), where it restricts NODAL to the future posterior side of the embryo. At this time, WNT3 signaling is activated and a positive feedback loop between BMP4 in the ExE and WNT3 and NODAL in the posterior epiblast leads to strong posterior activation of these signaling factors. After establishment of the anterior–posterior axis, gastrulation is initiated by the formation of the primitive streak (PS), where epiblast cells start to undergo an epithelial to mesenchymal transition under the influence of the signals at the posterior side of the epiblast. The mesenchymal cells in the PS leave the epiblast layer and migrate toward the anterior side of the embryo to give rise to the mesodermal layer (dark blue) between the epiblast and the VE. DE cells (dark green) leave the PS and migrate anteriorly together with the mesodermal cells in the distal half of the PS. DE cells will eventually integrate into the VE layer where the mixed cell population gives rise to the DE layer. Cells in the anterior epiblast will start to differentiate toward the NE by E7.5.

Next‐generation sequencing (NGS)‐based technologies such as chromatin immunoprecipitation‐sequencing (ChIP‐seq), assay for transposase‐accessible chromatin (ATAC)‐seq, or whole‐genome‐bisulfite‐seq (WBGS) in combination with transcriptional profiling by RNA‐seq now allow for detailed insights into chromatin state changes during cell lineage specification and differentiation. Especially when applied at single‐cell level, these technologies have greatly improved the knowledge of dynamic chromatin changes in different cell lineages (reviewed by [3, 7]). Epigenetic modifications are deposited by so‐called ‘writers’. These modifications are detected by ‘readers’ that also transduce the gene regulatory outcome and ‘erasers’ remove the modifications. Epigenetic modifiers include chromatin remodeling complexes (SWI/SNF, CHD complex, reviewed by [8]), DNA methyltransferases (DNMT proteins, reviewed by [9, 10]) and demethylases (TET proteins, [11, 12]), histone methyltransferases (HMTs) (SET1/MLL and PRC2 complexes, reviewed by [13]) and histone acetyltransferases (HATs) (p300/CBP, [14, 15]) and the corresponding ‘erasers’ of histone demethylases (KDM family, reviewed by [16]) and histone deacetylases (HDACs, reviewed by [17, 18]) (Table 1). Epigenetic modifiers are often ubiquitously expressed during early embryonic development [3, 19] and genetic deletion of these modifiers frequently result in pleiotropic phenotypes and embryonic lethality at different stages ranging from morula stage to perinatal lethality (as nicely reviewed in [3], Figs 1+2). Notably, phenotypes often are stage‐specific, suggesting some degree of specific requirements of epigenetic regulation during different developmental stages.

**Table 1:** Epigenetic modifiers covered in this review. The epigenetic modifiers discussed in the review are summarized. Alternative names, the catalytic function, the modification established by the modifiers and the complex they belong to are indicated.

Epigenetic modifier	Also known as	(Catalytic) function	Modification	Complex
MLL1	KMT2A	Histone methyltransferase	H3K4me3 (promoter)	MLL/COMPASS‐like
MLL2	KMT2B	Histone methyltransferase	H3K4me3 (promoter, bivalency)	MLL/COMPASS‐like
SETD1A	SET1A/KMT2F	Histone methyltransferase	Genome‐wide H3K4me2/3	SET1/COMPASS
MLL3	KMT2C	Histone methyltransferase	H3K4me1 (enhancer)	MLL/COMPASS‐like
MLL4	KMT2D	Histone methyltransferase	H3K4me1 (enhancer)	MLL/COMPASS‐like
EED		Activation of EZH2, binding to H3K27me3		PRC2
EZH1/2		Histone methyltransferase	H3K27me3	PRC2
RING1A/B	RNF1/2	E3 ubiquitin‐protein ligase	H2AK119ub	PRC1
p300	EP300	Histone acetyltransferase	H3K27ac, H3K9ac and others	
CBP	CREBBP	Histone acetyltransferase	H3K27ac, H3K9ac and others	
DNMT1/3A/3B		DNA methyltransferase	5mC	
TET1‐3		Methylcytosine dioxygenases	5hmC, 5fC, 5caC	
BRG1	SMARCA4	ATPase	Nucleosome rearrangement	SWI/SNF (also known as BAF)
BRM	SMARCA2	ATPase	Nucleosome rearrangement	SWI/SNF (also known as BAF)
CHD4		ATPase	Nucleosome rearrangement	NuRD

### Embryogenesis from the blastocyst to the gastrulation stage embryo

During the development from the blastocyst at embrinyonic day 3.5 (E3.5) to the E7.5 gastrulation stage embryo, the pluripotent cells of the embryo undergo a transition from naïve to formative and primed pluripotency to specification of the three germ layers mesoderm, definitive endoderm (DE) and neuroectoderm (NE) (reviewed by [1, 2]). The E3.5 blastocyst consists of two cell lineages, the outer extraembryonic trophectoderm (TE) lineage and the inner cell mass (ICM) (Fig. 1) (reviewed by [20]) that is characterized by the expression of TFs of the core pluripotency network *Pou5f1*, *Nanog*, and *Sox2* [21]. Until E5.5, the ICM differentiates into the epiblast that still retains core pluripotency factor expression but changed toward the formative pluripotency state (reviewed by [22]). At this stage, several signaling pathways are active in the epiblast, including NODAL, BMP, and FGF signaling, while WNT signaling activities are absent [23, 24, 25, 26, 27, 28]. Around E6.0 gastrulation starts which is indicated by the morphogenetic formation of the primitive streak (PS) at the prospective posterior side of the mouse embryo. BMP4 signals from the extraembryonic ectoderm and NODAL and WNT3 in the posterior epiblast form a positive feedback regulatory loop that results in strong posterior concentration of these signaling pathways [29]. NODAL and WNT signaling then initiate PS formation where cells undergo an epithelial–mesenchymal transition and ingress to form the nascent mesoderm and DE layers and become fate specified (Fig. 1) (reviewed by [30]). Early molecular markers for the onset of gastrulation are the T‐box TFs *Eomes* and *Tbxt* (*Brachyury*) in the cells giving rise to mesoderm and definitive endoderm (in short ME) [31, 32, 33]. In combination, EOMES and TBXT are indispensable for the transcription of all mesoderm and DE‐specific lineage genes, including lineage‐defining TFs such as *Mixl1, Foxa2, Lhx1, Gata6*, and *Mesp1* [34, 35]. The specification of mesoderm and DE is regulated by distinct TFs and is temporally and spatially separated, so that mesoderm is formed earlier and more proximally in the PS, shortly followed by DE cells that are generated more distally in the PS [36, 37, 38, 39, 40]. Cells of the anterior epiblast will differentiate toward NE between E7.0 and E7.5 as a result of the absence of ME‐inducing signaling activities. NE is thus formed by default when no ME is induced by early NE TFs including *Sox2* and *Pou3f1 (Oct6)* (Fig. 1) (reviewed by [1, 2]). Recent work has revealed some of the mechanisms that activate but also repress large gene expression programs by genome‐wide activities of TFs together with epigenetic modifiers, resulting in global changes in chromatin modifications and in transcriptional programs characteristic for different cell types and cell states (e.g., [37, 38, 41, 42]).

### *In vitro* models to study pluripotency and gastrulation

3D *in vitro* models of embryonic development are highly valuable tools to study early mammalian cell specification at molecular scale, including the analyses of chromatin dynamics. Embryoid systems help to overcome some of the limitations of experiments using early mouse embryo, which are difficult to manipulate and only generate limited material for analysis. Embryoid models are based on embryonic stem cells (ESCs) that were established by culturing the ICM of E3.5 blastocysts [43, 44]. ESCs exhibit pluripotency and have the ability to self‐renew and to contribute to the whole organism in chimera assays. ESCs can further self‐organize into embryoid bodies that differentiate into the three germ layers (reviewed by [45]). To date, mouse ESCs are mostly cultured in presence of LIF and 2 inhibitors (GSK3β and MEK inhibitors, resulting in WNT activation and MAPK/ERK inhibition) to maintain naïve ground‐state pluripotency resembling the ICM of the preimplantation embryo [46]. Naïve ESCs do not respond to germ layer inductive signals, thus these cells need to acquire a transition state called epiblast‐like cells (Epi‐LCs), which has been termed formative pluripotency [47, 48, 49, 50]. To recapitulate the state of the postimplantation epiblast, epiblast stem cells (EpiSCs) can be induced by prolonged culture of ESCs in presence of Activin A (ActA)/FGF or they can also be directly isolated from postimplantation embryos and cultured in the presence of ActA/FGF, and WNT inhibition and are in the primed pluripotent state [49, 51, 52, 53].w

Embryonic stem cells can be differentiated as embryoid bodies (EBs) to precursors of the three germ layers [45, 54]. Here, ESCs are seeded in serum‐free medium‐forming cell aggregates (EBs). The differentiation can be driven to NE or ME by additional signaling cues like retinoic acid (RA), NODAL (ActA) or WNT [55, 56, 57]. To investigate spatiotemporal development and the interplay of extraembryonic‐ and embryonic development, new *in vitro* cultures such as gastruloids, blastoids, ETS, and ETX (including iETX or EiTiX) embryoids (see abbreviations) have been developed [58, 59, 60, 61, 62, 63, 64]. Gastruloids are generated by self‐assembled ESCs and reveal the spatiotemporal development of gastrulation and somitogenesis [58]. In blastoids, ETS and ETX models ESCs are combined with cultured extraembryonic trophoblast stem cells and/or primitive endoderm cells to recapitulate embryonic development more closely. Here also the interplay between embryonic and extraembryonic tissues can be investigated.

In this review, we provide an overview of the current understanding of some of the epigenetic mechanisms that contribute to exit of pluripotency and lineage specification in early embryonic mouse development. We describe the dynamic chromatin states at CREs regulating lineage‐specific gene expression. Further we delineate the roles of chromatin modifiers in the establishment of these chromatin states and discuss questions of lineage‐specific functions of certain chromatin modifications. Here, we especially focus on DNA methylation, the bivalent chromatin state at promoters, the role of activating histone modifications at promoters and enhancers, and the dynamic regulation of chromatin accessibility. We discuss the role of TFs for cell lineage‐specific recruitment of epigenetic modifiers and the epigenetic differences between NE and ME lineage specification. Finally, we provide a provisional model that explains the sequence of events for activation of enhancers for the transcription of cell‐type‐specific developmental genes.

## Cell lineage‐specific gene expression correlates with dynamic changes at enhancers

Mammalian gene transcription is controlled by the concerted interactions of TFs, epigenetic modifiers, and other cofactors at the chromatin of cis‐regulatory elements (CREs), that can be broadly classified into enhancers and promoters. During embryonic cell lineage specification, the chromatin at CREs is dynamically regulated by epigenetic modifications that impact the ability of transcriptional regulators to interact with CREs and thus regulate lineage‐specific gene expression (reviewed by [5, 6]).

**Fig. 2:**
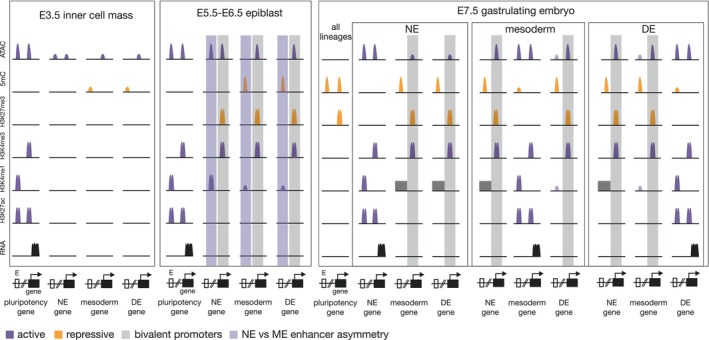
Chromatin dynamics at enhancers and promoters during pluripotency exit and lineage specification in the mouse embryo. Pluripotency genes of future epiblast cells in the inner cell mass of E3.5 embryos show active chromatin marks at promoter and enhancer regions and are consequently accessible and expressed. Active marks (H3K4me3, H3K4me1, and H3K27ac) show the characteristic double peak, as regions are nucleosome‐depleted. Cis‐regulatory elements (CREs) of neuroectoderm (NE), mesoderm and definitive endoderm (DE) lineage‐associated genes lack active histone marks and H3K27me3 and genes are not expressed. At E3.5, overall methylation levels are low. In the formative epiblast state (E5.5‐E6.5 epiblast), pluripotency genes are still actively transcribed and there is no change in chromatin state. At NE, mesoderm and DE promoters H3K27me3 is placed by E5.5 and H3K4me3 by E6.5 establishing bivalency (light gray bars). While NE enhancers are demethylated and in an accessible chromatin state, mesoderm and DE enhancer are closed and methylated (light purple bars). Before activation of mesoderm and DE enhancers, they are in a primed state characterized by a single H3K4me1 peak. If this state is only established after demethylation has not been investigated. During lineage specification in the gastrulation stage embryo at E7.5 naïve pluripotency genes are downregulated. Active marks at pluripotency enhancers and promoters are lost, promoters are methylated and repressed by polycomb‐mediated H3K27me3. As the NE enhancer landscape is already accessible and demethylated in pluripotent cells, NE lineage specification in the anterior epiblast is initiated by activating histone marks at NE enhancers and promoters. In contrast, mesoderm and DE enhancers become demethylated and accessible in a lineage‐specific manner. As enhancers become accessible the primed small H3K4me1 peak transitions to a double peak and is accompanied by H3K27ac. Whether the H3K4me1 priming at DE enhancers is maintained as a single peak or if accessibility and a H3K4me1 double peak is established in the mesoderm lineage (and vice versa) was not analyzed (depicted by a transparent single H3K4me1 and accessibility peak in the DE and mesoderm lineages). If H3K4me1 priming of mesoderm and DE enhancers is removed in the NE lineage and if accessibility and H3K4me1 of NE enhancers is removed in mesoderm and DE lineages has not been analyzed (depicted by a dark gray box). NE gene enhancers become methylated and closed upon mesoderm and DE differentiation. Bivalency at promoters is resolved in the respective NE, mesoderm, and DE differentiating cells. This figure is based mainly on data from [82, 83, 84, 85].

**Fig. 3:**
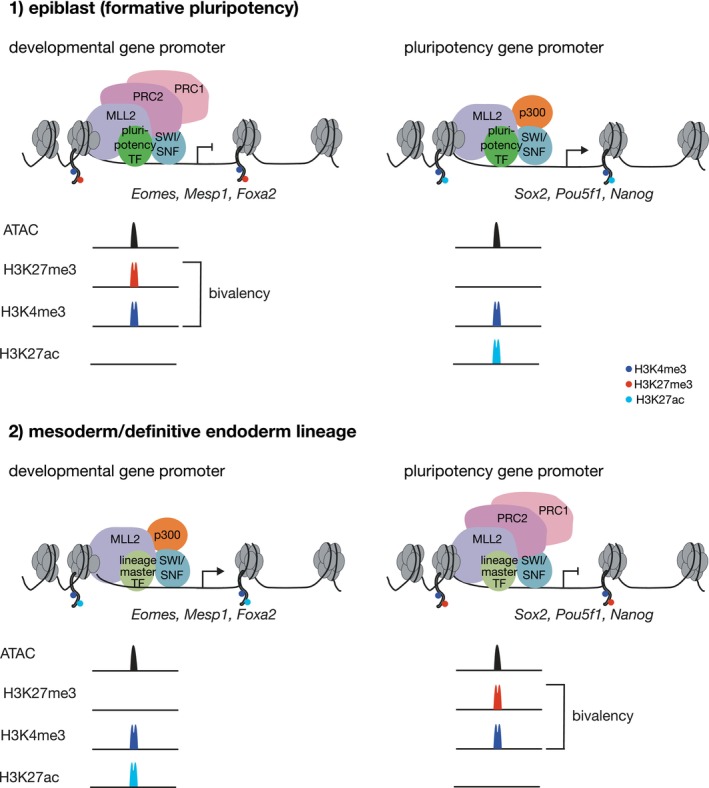
Promoter bivalency at developmental and pluripotency genes. Shortly before gastrulation initiation in the mouse embryo, pluripotent epiblast cells show bivalency at developmental gene promoters (*Eomes*, *Mesp1*, and *Foxa2* as example genes) (1). Bivalency is characterized by the simultaneous presence of the activating H3K4me3 and the repressive H3K27me3 histone marks on histones around the promoter region. These marks are established by MLL2 (H3K4me3) and the polycomb repressive complex 2 (PRC2) (H3K27me3). Genes possessing bivalent histone marks show no or very low expression. PRC1 is frequently present at the same sites as PRC2 and also places a repressive histone mark (H2aK119ub, not shown). At pluripotency gene promoters (*Sox2*, *Pou5f1*, and *Nanog* as example genes), histones are marked by H3K4me3 and H3K27ac (catalyzed by p300) and these genes are actively transcribed. Pluripotency and developmental gene promoters are accessible (peak in ATAC track), which is established by the SWI/SNF complex. Both active pluripotency gene promoters as well as inactive bivalent developmental gene promoters are bound by pluripotency transcription factors (TFs) [247] (1). Upon differentiation toward mesoderm and definitive endoderm lineages, developmental gene promoters are bound by lineage specifying master TFs, leading to dissociation of PRC2/PRC1 and binding of p300, establishment of H3K27ac and active transcription (2). At the same time lineage master TFs bind pluripotency gene promoters, PRC1/PRC2 are recruited to these promoters and bivalency is established and pluripotency genes are no longer transcribed (2). Long‐term silencing is later mediated by H3K27me3 in absence of H3K4me3 and by DNA methylation. Enzymes removing histone marks (histone demethylases and histone deacetylases) are not shown here.

*In vitro* studies showed that promoters and enhancers are characterized by chromatin marks of tri‐ (H3K4me3) and mono‐methylation (H3K4me1) at lysine 4 of histone 3, respectively [65, 66]. H3K4me3 is predominantly placed by the histone methyltransferases (HMTs) MLL1/MLL2 (also known as histone lysine N‐methyltransferase KMT2A/B) ([67], reviewed by [68]) and H3K4me1 by MLL3/MLL4 (also known as KMT2C/D) ([69], reviewed by [70]) (Table 1). CREs of actively transcribed genes additionally show acetylation at lysine 27 of histone 3 (H3K27ac) and have accessible nucleosome‐depleted chromatin [65, 66, 71] (Fig. 2). H3K27 acetylation is catalyzed by the histone acetyltransferases (HATs) CBP and/or p300 [14, 15], and chromatin accessibility is established by ATP‐dependent chromatin remodeling complexes, such as the SWI/SNF (BAF) complex and others [8]. Enhancers have also been identified in a primed state, which is characterized by a single H3K4me1 peak as these regions are not nucleosome‐depleted and thus have low chromatin accessibility [65, 72, 73, 74] (Fig. 2, E5.5‐E6.5 epiblast). This initial priming of enhancers by H3K4me1 is regulated by pioneer TFs, that can bind to their DNA‐binding motif when DNA is nucleosome‐engaged [75] (reviewed by [72]) and has also been observed during ESC differentiation before initiation of ME formation [42]. CREs of developmentally regulated genes additionally show global differences in DNA methylation patterns. Enhancers typically possess intermediate levels of DNA methylation described as lowly (or intermediated) methylated regions (LMRs) [76, 77]. Promoters of developmental/tissue‐specific and housekeeping genes are in most cases in close proximity to or contain cytosine‐phosphate‐guanine dinucleotide (CpG)‐rich regions, so‐called CpG islands. These CpG islands are mostly unmethylated [78]. During pluripotency exit and gastrulation onset, these unmethylated promoters are often found in the so‐called bivalent chromatin state. Bivalency is a pre‐activation state, indicated by the co‐occurrence of the active chromatin mark H3K4me3, and the repressive mark tri‐methylation at lysine 27 of histone 3 (H3K27me3), which is deposited by HMT activity of the polycomb repressive complex 2 (PRC2) (reviewed by [79]) (Table 1, Fig. 2 light gray bars, Fig. 3).

Several studies analyzed the epigenetic states of the genome‐wide CRE landscapes in mouse embryos before and during gastrulation by single‐cell multiome sequencing or tissue micro‐dissection [77, 80, 81, 82, 83, 84, 85]. These analyses confirmed previous *in vitro*‐derived observations that promoters and the transcription start site (TSS) are largely hypomethylated [77, 83, 84]. Expectedly, histone marks for active promoters (H3K4me3, H3K27ac) and enhancers (H3K4me1, H3K27ac) positively correlate with gene expression in micro‐dissected tissues of mouse embryos from E6.5 to E7.5 [83, 85]. The correlation of active histone marks with transcription is generally stronger at TSS‐flanking regions than at gene‐distal regions [83, 85]. However, activation of gene expression during differentiation from E4.5 to E7.5 did mostly not correlate with dynamic methylation and accessibility changes at promoters. Promoters were in an accessible and demethylated state in stages before activation of gene expression occurred [84] (Fig. 2). Cell‐type‐specific enhancers on the other hand show changes of accessibility and of DNA methylation that correlate with lineage‐specific gene expression at the same stage [77, 84]. Accordingly, during mouse gastrulation the dynamic changes of the enhancer mark H3K4me1 exceed those of the promoter mark H3K4me3, emphasizing that dynamic enhancer modifications reflect lineage specification [83]. In summary, embryo and ESC differentiation studies demonstrated that cell lineage‐specific gene expression is predominantly accompanied by dynamic changes at enhancers and not at promoters [42, 77, 83, 84, 86]. Nevertheless, the correct regulation of the epigenetic state of promoters is essential for gastrulation as discussed below.

## DNA methylation as epigenetic barrier against lineage‐aberrant gene expression

DNA methylation serves as long‐term repressive mark that is often associated with roles in silencing of larger genomic regions or gene clusters, such as X chromosome inactivation, silencing of repetitive, transposable elements, and genomic imprinting. More recently additional regulatory roles of dynamic DNA methylation during the specification of different cell lineages were revealed, such as during the exit of pluripotency at gastrulation and embryonic cell lineage specification. Global levels of DNA methylation follow a dynamic pattern in the course of embryonic development [87, 88]. After fertilization, DNA methylation patterns in the zygote are largely erased and methylation levels are globally reduced. Global levels of methylation levels start to rise again following implantation (E4.5–6.5) when methylation patterns become re‐established. These changes in methylation are accompanied by high expression levels of the *de novo* DNA methyltransferase 3b (*Dnmt3b*) in the epiblast [89].

### DNA methylation functions as repressive mechanism of alternative lineages and pluripotency genes in the epiblast

Embryonic and extraembryonic cell lineages acquire unique DNA methylation profiles, where extraembryonic lineages exhibit globally reduced methylation levels [90]. Interestingly, the genetic deletion of maintenance *Dnmt1* results in the aberrant expression of genes of the extraembryonic trophectoderm‐lineage, such as the Ets transcription factor *Elf5*, in the embryonic epiblast [91]. In addition, CpG island promoters of embryonic lineage genes are hypermethylated in extraembryonic tissues [92], and VE genes show hypermethylation in the E6.5 epiblast [77]. These data show that DNA methylation serves as long‐term epigenetic barrier that safeguards embryonic and extraembryonic cell identities by silencing of genes of alternative lineages.

CpG island containing promoters of housekeeping and developmental genes remain as largely unmethylated regions (reviewed by [78, 93]) and are protected from methylation by the activating H3K4me3 mark deposited by MLL2 [94]. However, a small subset of CpG island promoters become methylated after implantation, which comprise promoter CpG islands of germline‐ and pluripotency‐associated genes [77, 88, 95] that are normally repressed during differentiation toward somatic lineages. This is in line with the incomplete repression of the pluripotency‐associated gene regulatory network (GRN) in differentiating *Dnmt1,3a,3b*‐deficient ESCs [96]. Subsequent G9A‐mediated methylation at lysine 9 of histone 3 (H3K9) is suggested to initiate long‐term silencing of these genes [95, 97]. In summary, lineage‐specific patterns of DNA methylation in large parts serve as epigenetic barrier between extraembryonic and embryonic lineages, and play roles in the repression of the germline/pluripotency‐associated gene networks.

### Demethylating TET enzymes are required to establish permissive enhancer landscapes for the process of gastrulation

More recently, genetic deletions of the *Tet* enzymes have started to reveal the nature of the fine‐tuned regulatory functions by the DNA methylation and DNA demethylation cycle during embryogenesis. TET enzymes act as the demethylases that remove CpG methylation by catalyzing steps of oxidation of 5‐methylcytosine (5mC) that are followed by a base excision repair mechanism that results in an unmethylated cytosine [11, 12, 98, 99]. Of the three *Tet* enzymes, *Tet1* and *Tet2*, are highly expressed in the pluripotent cells of the ICM (reviewed by [100]) where *Tet1* expression is maintained until E6.5‐E7.5, while *Tet2* is downregulated shortly after implantation (E4.5) [101]. *Tet3* starts to be weakly expressed at E8.5 in head folds and neural tubes and around E9.5–10.5 expression of all *Tet* genes is restricted to the developing brain.

During cell lineage specification at gastrulation, mesoderm‐ and DE‐specific distal enhancers become demethylated in a tissue‐specific manner [84] (Fig. 2, E7.5 gastrulating embryo 5mC) and *Tet1,2,3*‐deficiency results in hypermethylation at predominantly distal enhancer regions [84, 86, 102], thus suggesting that *Tet* enzymes play roles in cell‐type‐specific enhancer regulation. Additionally, *Tet1,2,3*‐deficient ESCs only poorly differentiate *in vitro* [103], and *Tet* triple mutant (TKO) embryos show strong phenotypes around gastrulation stage [104, 105], predominantly failing to form ectoderm and mesoderm cell types [102, 104, 105]. Analysis of *Tet*‐TKO mutant embryos and ESCs revealed increased NODAL and WNT signaling levels as a result from hypermethylated CREs of the NODAL (*Lefty1/2*) and WNT (*Sfrp4*) pathway inhibitors [104, 105]. Elegant studies of chimeras composed of *Tet*‐TKO and wild‐type cells showed that the presence of wild‐type cells could rescue major parts of the *Tet*‐TKO phenotype, supposedly by providing an intact signaling environment [102]. Despite partial hypermethylation at bivalently marked H3K4me3/H3K27me3‐promoter sites and extensive hypermethylation at enhancers, the *Tet*‐TKO cells in mosaic chimeric embryos show remarkably few changes in their gene expression signatures [102], similar to previously observed modest correlation of DNA methylation and gene expression changes in differentiating *Tet1,2,3*‐deficient cells [86, 102, 103]. These rather modest correlations might explain the delayed phenotypes of mutant embryos for *Dnmt3* and *Tets* when compared to their stage‐specific expression as more obvious phenotypes occur after their peak of expression. Dynamic changes in DNA methylation levels might not immediately regulate gene expression, but could also mediate alternative functions. These could lie in the generation of a permissive chromatin landscape that is required during later developmental stages for lineage specification, as this was demonstrated for lineage priming functions of TET1 and DNMT3B in pluripotent ESCs [106, 107]. In these studies, TET1 and DNMT3B are required early during the transition from naïve pluripotency to primed epiblast to ensure subsequent correct commitment toward NE or ME, respectively. In line, the oxidized derivatives of 5‐methylcytosine, such as 5‐hydroxy‐, 5‐formyl, 5‐carboxylcytosine (5hmC,5fC,5caC), might have distinct epigenetic functions as, for example, recruiting TFs and other epigenetic modifiers (reviewed by [108]), which implies the requirement of DNA methylation turnover at specific sites at a specific time (reviewed by [109]).

In summary, the demethylation by *Tet* enzymes has functions in fine‐tuning of gene expression programs that may provide robustness of lineage decisions by regulating the signaling environment and establishing a permissive enhancer landscape rather than instructing cell‐type‐specific gene programs.

## Polycomb group proteins establish bivalency at developmental promoters

Similar to DNA methylation, the repressive chromatin marks deposited by polycomb repressive complexes (PRCs) are involved in long‐term silencing mechanisms such as genomic imprinting, X chromosome inactivation, and separation of embryonic and extraembryonic tissues (e.g., [94, 110], and reviewed by [111]). However, PRCs are also responsible for the repressive mark at bivalent promoters and are therefore required for bivalency (reviewed by [111, 112, 113]) (Fig. 3). The investigation of bivalency at developmental promoters has shown that correct epigenetic regulation of promoter elements is crucial for appropriate transition from pluripotency to lineage specification.

### Bivalency: the simultaneous presence of active and repressive histone marks at promoters and enhancers

Studies in ESCs have identified a chromatin state at promoters, which has been termed bivalent as it is associated with an active (H3K4me3) and repressive (H3K27me3) mark simultaneously [73, 114, 115, 116, 117] (reviewed by [79]) (Fig. 2, E5.5‐E6.5 epiblast and E7.5 embryo light gray bars: NE, mesoderm and DE promoters, Fig. 3). Bivalent promotors are often associated to developmental genes and maintain gene silencing but are postulated to ensure rapid gene activation during differentiation. Upon lineage specification, bivalency resolves and developmental genes become transcriptionally active or inactive [79, 115, 116]. Additional functions of bivalency may lie in the prevention of permanent promoter‐silencing by DNA methylation mediated by the active mark H3K4me3 [118]. In the gastrulating mouse embryo, the changes in gene expression are regulated by transitions from bivalent to active promoters and from active to bivalent rather than acquiring a fully repressed promoter state [83]. In contrast to ESCs, bivalency at developmentally regulated promoters in the embryo is established relatively late in the E6.5 epiblast shortly preceding gastrulation onset. H3K27me3 at promoters is present in the E5.5 epiblast, but the co‐occurrence of H3K4me3 at promotors is only established around E6.5 [85] and is not present in preimplantation embryos [81, 85] (Fig. 2, light gray bars). Therefore, the bivalent promoter state of ESCs likely represents a feature of the formative pluripotent epiblast in the embryo.

In addition to bivalency at promoters, a similar bivalent chromatin state at enhancers (sometimes also called poised enhancers) is characterized by the simultaneous presence of the enhancer mark H3K4me1 and the repressive mark H3K27me3 at the same nucleosomes [119, 120]. Bivalent, poised enhancers are mostly nucleosome‐depleted, DNA‐hypomethylated, and bound by the histone acetyltransferase p300 and the SWI/SNF (BAF) nucleosome remodeling complex that contains the central ATPase BRG1 (SMARCA4) ([119, 120] reviewed by [121]). Bivalent enhancers are also often associated with developmentally regulated genes. In addition to work in ESCs, the bivalent enhancer signature was also observed in the embryo [83], but not yet confirmed on a single‐cell level. The precise role of enhancer bivalency, its regulation, and functions during cell lineage differentiation will require further investigations.

### Polycomb repressive complexes during pluripotency exit

What are the epigenetic modifiers that establish bivalent chromatin marks both at promoters and enhancers? The active promoter mark H3K4me3 is deposited by MLL2, of the MLL1/MLL2 Compass‐like complex, that belongs to the trithorax protein group [122] (Table 1, Fig. 3). The related MLL4 predominantly catalyzed H3K4me1 at enhancers [123], while MLL3 acts redundantly *in vitro* [124, 125]. The repressive histone mark H3K27me3 is catalyzed by the SET‐domain containing methyltransferase subunits EZH1 and EZH2 of the polycomb repressive complex 2 (PRC2) [114, 115, 117] (Table 1, Fig. 3).

Two different PRCs exist, namely PRC1 that functions as a histone ubiquitin ligase to mono‐ubiquitinate lysine 119 at histone 2A (H2AK119) [126, 127] and the histone methyl transferase complex PRC2 that mediates tri‐methylation of H3K27me3. Although PRC2 deposits the repressive mark of bivalency, PRC1 is mostly also detected at PRC2‐occupied sites (see below and reviewed by [111]). The core subunits of PRC1 are RING1A or RING1B and one of six polycomb group RINGfinger domain proteins (PCGF1‐6). The core subunits of PRC2 are EED, SUZ12, EZH2/EZH1, and RBBP4/RBBP7 (Table 1). Both complexes exist in different variants and PRC1 has canonical and noncanonical complex variants (reviewed by [111, 113, 128]). Studies in ESCs have shown that PRC1 and PRC2 are bound to promoters of developmental regulator genes ([129, 130, 131, 132]) (Fig. 3). In this context, they negatively regulate transcription of these genes (see below). PRC1 core subunit RING1B (also RNF2) and PRC2 core subunits EED and SUZ12 binding patterns overlap strongly suggesting combined functions for bivalency [129, 130, 132, 133]. While previous views supported successive binding of PRC complexes to target sites, their recruitment is at least partially independent from each other, and both PRC1 and PRC2 associate with target sites in the absence of the other (reviewed by [134]).

The genetic deletions of core components of both PRCs in pluripotent cells result in an increased expression of cell lineage marker genes, suggesting roles of polycomb for the repression of cell lineage specification programs in the epiblast [114, 129, 133, 135, 136, 137]. For example, the single deletion of *Ring1b* or both *Ring1b/Eed* in pluripotent ESCs made them prone to differentiate during ESC culture [133, 135]. Similarly, the deletion of both *Ring1a* and *Ring1b* leads to the loss of typical ESC characteristics, including morphology, reduced proliferation, and the upregulation of developmental genes [138]. However, deletion of core components of either PRC1 (*Ring1b*) and PRC2 (*Eed, Suz12, Ezh2*) or both *Ring1b/Eed* does not compromise self‐renewal of ESCs, suggesting that PRCs are not essential for ESC maintenance in pluripotency permissive conditions [133, 135, 136, 139, 140].

### Polycomb repressive complexes during gastrulation and lineage specification

Mouse embryos deficient for polycomb group (PcG) protein members develop past early postimplantation stages, preceding E5.5 (reviewed by [128]), further indicating that PRCs are not crucial during naïve pluripotency. The loss‐of‐function mutants of core components of PRC2 [*Eed*, *Ezh2*, and *Suz12*, but not *Ezh1* (which is not expressed early)] and PRC1 [*Ring1b* but not *Ring1a* (later phenotype, homeotic transformation)] lead to phenotypes at gastrulation stage demonstrating their crucial functions for this process [141, 142, 143, 144, 145]. Phenotypes in *Eed* mutants served as paradigm for PRC2 functions during gastrulation. Single‐cell RNA‐seq and morphological studies demonstrate relatively unperturbed development until E6.5. After E6.5, following gastrulation onset, *Eed*‐deficient embryos show accumulation of extraembryonic mesoderm (ExM) and primordial germ cells (PGCs) in the posterior PS region. ExM and PGCs are relatively overrepresented at the cost of specification of other mesoderm derivatives and NE [94, 142]. In addition, the proper sequence of *Hox* gene cluster activation is disturbed and pluripotency genes are not properly downregulated [94]. The enhanced generation of PGCs may result from the insufficient repression of pluripotency‐ and PCG‐associated genes. This suggests that this phenotype is caused by a lack of active to bivalent transition to silence pluripotency and PCG programs in the germline progenitors. Furthermore, the overrepresented ExM is the lineage generated first during gastrulation. Therefore, it is not entirely resolved if cell misspecification after *Eed* deletions come from severe developmental delays caused by pluripotency and PGC programs, or if PRC2 is indeed specifically required for the regulation of the cell lineage‐specific gene programs of the missing tissues. These phenotypes are at least partially recapitulated in *Ring1b(Rnf2)*‐deficient embryos, arguing for converging functions of PRC1 and PRC2 during lineage specification [94, 145].

Studies in differentiating pluripotent cells mutant for various core and associated PcG components resulted in an heterogenous view on the requirement for the regulation of cell‐type‐specific gene programs. The requirement of PcG proteins for induction of specific lineages such as mesoderm, NE, or all embryonic lineages combined have been described (e.g., [135, 146, 147]). Other studies suggest that lack of either PRC1 or PRC2 still allow differentiation toward all three germ layers [133, 148, 149, 150] and that only combined lack of PRC1 and PRC2 result in failed gastrulation differentiation [133]. The seemingly conflicting results of high variability in differentiation defects suggest that PRCs rather have general roles in safeguarding specification programs. These functions may lie in the repression of alternative cell lineage and pluripotency gene programs, rather than playing specific functions for the induction of certain cell lineages (e.g., [94, 120, 130, 133, 136, 147, 151, 152, 153]).

In summary, bivalency at promoters of developmental and pluripotency/PCG genes is established by PRC2 (repressive H2K27me3) and MLL2 (active H3K4me3). The repressive mark placed by PRC2 functions in keeping lineage‐specific genes silenced before gastrulation and in the repression of pluripotency and PGC gene programs during differentiation of NE and ME (Fig. 3). In addition, alternative lineage genes are also repressed by PcG proteins. Several open questions concerning the regulation by PRCs during gastrulation stages of mammalian development remain. For example, it remains unclear how PRCs are recruited in dynamic and specific fashion to CREs of developmental genes to repress alternative gene programs in a cell‐type‐restricted manner.

## Are active marks required for gene expression?

*In vitro* models using ESCs established the epigenetic states of active promoter (H3K4me3 and H3K27ac) and enhancer regions (H3K4me1 and H3K27ac) ([65, 66, 67, 69, 71] and reviewed by [7, 68, 70]). Although lineage‐specific gene expression is mostly accompanied by epigenetic changes at enhancers, gene expression showed higher positive correlation with active promoter marks in TSS‐flanking regions *in vivo* [83, 85].

### Redundantly acting histone methyltransferases (HMTs) of the promoter mark ensure correct regulation of gene transcription

The high‐positive correlation of lineage‐specific gene expression with active promoter marks suggests a requirement of H3K4me3 HMTs in lineage specification. As key developmental gene promoters are in a bivalent state in ESCs and the pregastrulation epiblast, the two‐histone methyltransferases predominantly depositing H3K4me3, namely MLL2 and SETD1A, are of most interest. In ESCs [122, 154] and in E6.5 epiblast cells [85], MLL2 is the main HMT depositing H3K4me3 at bivalent sites. Deficiency of *Mll2* does not affect initiation of differentiation, but causes delays in ectoderm and mesoderm differentiation *in vitro* [155]. This delay is most likely caused by increased H3K27me3 at bivalent sites as this has been shown *in vitro* for acute depletion of *Mll2* which further causes loss of chromatin accessibility and reorganization of 3D chromatin structure [156]. However, lineage‐specific functions are not observed in *Mll2* ^*−/−*^ embryos [157]. Here, *Mll2* deficiency results in embryonic lethality by E10.5 suggested to be caused by increased apoptosis rates. Interestingly, H3K4me3 ChIP‐seq analysis of dissected head tissues of E8.5 *Mll2*‐deficient embryos revealed global loss of H3K4me3 at bivalent sites [85]. Still, changes in gene expression of the associated genes were rather subtle and H3K4me3 was partially maintained at some bivalent gene promoters. This suggests that MLL2‐deposited H3K4me3 at bivalent sites may be dispensable for gene expression of some developmental genes, but mediates robustness of lineage decisions during gastrulation as other HMTs most likely act redundantly. One of the studies in ESCs proposed SETD1A as redundant‐acting H3K4me3 depositor at bivalent gene promoters during differentiation *in vitro* [158]. *Setd1a*‐deficient mice show the earliest and most severe phenotype of H3K4 HMT mutant embryos with embryonic lethality by E7.5 and increased apoptosis as has been observed for *Mll2*‐deficient mice [159]. Attempts to generate *Setd1a* ^*−/−*^ ESCs from blastocysts were not successful [159]. The generation of ESCs harboring catalytically inactive SETD1A allowed the evaluation of the requirement of H3K4me3 deposition by SETD1A in pluripotency and lineage specification [158]. Interestingly, SETD1A catalytic function is not essential to maintain pluripotency, but only required during differentiation of mainly mesoderm and NE lineages as mesoderm genes were not induced and NE differentiation was defective by day 3 of differentiation [158]. To conclude, bivalency is established prior to gastrulation by depositing H3K4me3 at bivalent sites in E6.5 epiblast cells, which primes developmental genes for rapid activation upon lineage specification [85]. If this priming by H3K4me3 is essential for subsequent gene expression is not clear to date. Redundancy of H3K4 methyltransferases [85, 154, 158, 160], which results in robustness of H3K4me3 deposition, hampers a detailed mechanistic analysis. However, several studies have implicated the requirement of H3K4me3 in gene expression as it recruits the TFIID basal transcription factor complex [161, 162] and RNA polymerase (RNA Pol) II [160] and is involved in RNA Pol II pause release [163].

### The enhancer mark H3K4me1 characterizes cell identity

As lineage‐specific gene expression is regulated by enhancer CREs, the function of H3K4me1 in lineage specification was investigated by studies on MLL3/4. MLL4 has been shown to be the predominant HMT for H3K4me1 deposition at enhancer regions [123], however, MLL3 partially compensates for the loss of MLL4 [124, 125]. Current studies focus on the question if H3K4me1 is required for lineage‐specific gene expression or if it can be solely used to identify cell‐type‐specific enhancer regions. In ESCs, MLL4 binds to active enhancer regions of pluripotent genes. However, *Mll3/4*‐deficient cells maintain pluripotency [123] suggesting that either MLL3/4‐mediated H3K4me1 is not required for maintaining gene expression or that another H3K4me1 HMT acts redundantly. Upon differentiation, *Mll3/4*‐double deficient ESCs fail to induce mesoderm and endoderm marker genes and fail to give rise to any germ layer derivatives in teratoma assays [123]. Here, MLL3/4 are required to establish active enhancer regions, but this was suggested to be mediated by the recruitment of the histone acetyltransferase p300 by MLL4 and not H3K4me1. Further dissection of the catalytic and noncatalytic functions of *Mll3/4 in vitro* [164] showed that only full‐length deletions result in strong reduction of the active mark H3K27ac and reduced gene expression of *Mll3/4*‐associated genes. The catalytically dead MLL3/4 was still capable of recruiting RNA Pol II and thus showed only minor changes in gene expression [164]. *In vivo*, double‐knock‐in of catalytically dead *Mll3/4* in mice caused embryonic lethality around E6.5 [125], which was caused by MLL3/4 functions in extraembryonic tissues. While the catalytic activity of MLL3/4 was dispensable for germ layer specification, more detailed analysis *in vitro* has shown that MLL3/4 catalytic activity is required for proper differentiation of extraembryonic endoderm (ExEn). Upon *in vitro* ExEn differentiation, binding of the master transcription factor GATA6 to enhancers is lost in MLL3/4 catalytically dead cells, which might pinpoint to a premarking function of GATA6‐binding sites by H3K4me1. To conclude, H3K4me1 can be utilized to identify putative enhancers of cell identity. While MLL3/4 has been shown to be crucial for establishing active enhancers upon lineage specification, H3K4me1 is suggested to be a consequence but not causal for enhancer activation in embryonic lineages during gastrulation. Of note, bulk H3K4me1 is not completely lost in *Mll3/4* double knockout and catalytically dead ESCs [125] implying the involvement of further H3K4me1 methyltransferases in enhancer regulation as shown for MLL2 [165]. Besides, the catalytic activity of MLL3/4 seems to be required for the establishment of enhancer–promoter interactions during ESC to neural progenitor cell differentiation [165].

### The contribution of the active histone mark H3K27ac on gene transcription

When promoter and enhancer regions are in an active state, they are further characterized by H3K27ac [71], that is placed by the histone acetyltransferases p300/CBP [15] (Table 1). *In vivo*, there is a high correlation between gene expression and H3K27ac [83], which can be attributed to the function of p300 as catalytically dead‐Cas9 (dCas9) targeting of p300 to promoter and enhancer sites leads to gene activation *in vitro* [166]. As for H3K4me1, it is of great interest if H3K27ac itself is required for gene activation or if it is a ‘byproduct’ of p300 binding. Chemical inhibition of p300/CBP catalytic activity with preserved chromatin binding leads to reduced chromatin accessibility at enhancers and promoters caused by impaired BRD4, TFIID, and RNA Pol II recruitment *in vitro* [167] emphasizing the importance of the H3K27ac mark itself. However, abolishment of H3K27ac modification on the enhancer‐enriched histone H3.3 by mutation of lysine 27 to arginine in ESCs showed only minor changes in transcriptome, also chromatin accessibility, H3K4me1, and other histone lysine residue acetylations were mostly unchanged [168]. Compensatory functions of H3.1/2 were excluded as another study generated ESCs harboring lysine 27 to arginine mutations on all alleles of H3.1. H3.2 and H3.3 [169]. This study further supported that loss of H3K27ac shows only minor changes in gene expression in a static system and during ESC‐to‐EpiLC transition. Activation of gene expression in these mutant ESCs were rather attributed to the loss of the repressive mark H3K27me3. Thus, and in opposite to promoter regions, H3K27ac at enhancer sites seems to be dispensable for gene expression in a static situation. It remains undefined if H3K27ac at enhancer regions is crucial for gene expression upon differentiation or if acetylation of other lysine residues may compensate for enhancer activation.

As suggested for H3K4me1 and GATA6, H3K27ac could premark enhancer regions of more differentiated cells as analysis of H3K27ac upon early and late mouse development *in vivo* revealed a premarking of organogenesis‐related genes with H3K27ac upon gastrulation [83]. Although the expression levels of these organogenesis‐related genes were low during gastrulation, they increased at later developmental stages.

To conclude, the activating histone marks, especially the enhancer marks can be used to identify cell identity‐specific enhancers as MLL3/4 and p300/CBP bind to active CREs and catalyze the histone modifications. Activating histone marks are potentially not individually required for lineage‐specific gene expression. However, the regulation of activating histone marks seems to be quite robust and redundant mechanisms might be the cause for the absence of phenotypes. The analysis of catalytically inhibited HMTs and HATs have also shown that these epigenetic modifiers have functions beyond their methyl‐ or acetyltransferase activities. Other roles seem to be the recruitment of factors/complexes involved in subsequent activation of gene expression, such as RNA Pol II. This suggests that the whole process is highly redundantly regulated and that absence of a single modification will still allow gene expression to be activated. Only disintegration of the regulatory complexes or multiple effects might therefore severely affect gene expression.

## Recruitment of chromatin remodeling complexes by lineage‐specific transcription factors

In the embryo and in ESCs, a network of pluripotency TFs governs the pluripotent state by regulating a gene network (reviewed by [21]). During subsequent lineage specifications, master TFs are crucial for induction of lineage‐specific gene programs (e.g., [34, 39, 41]). For pluripotency and some lineage‐specific TFs, it was shown that they functionally interact with the SWI/SNF chromatin remodeling complex (e.g., [42, 170, 171]). Chromatin remodelers are complexes that use the energy from ATP hydrolysis to change the nucleosome distribution on the chromatin. Four different classes of chromatin remodeling complexes exist, the SWI/SNF (or BAF), the ISWI, the CHD (including NuRD complex), and the Ino80 complexes. Chromatin remodeling complexes are required to modulate chromatin accessibility at enhancers and promoters to make them available for binding of TFs and other factors regulating gene expression (reviewed by [8, 172]). They are required to maintain pluripotency but also to allow rapid activation of developmental genes during differentiation (reviewed by [172, 173]). During mouse development, all four remodeling complexes are crucial throughout development. They are all required for the survival of mouse embryos around the blastocyst or early implantation stage (SWI/SNF: [174], ISWI [175, 176], CHD/NuRD [177, 178], and INO80 [179, 180, 181, 182]). During pluripotency and subsequent lineage specification at gastrulation, important roles for the SWI/SNF (BAF) complex and the CHD‐containing NuRD complex have been described (reviewed by [172]).

### The SWI/SNF complex is recruited by lineage‐specific TFs to establish enhancer accessibility

For the SWI/SNF complex, it is well documented that its function during pluripotency and lineage specification relies on recruitment by lineage‐specific TFs. The SWI/SNF complex slides and evicts nucleosomes from its target sites and therefore establishes open chromatin (reviewed by [8]). SWI/SNF contains either the ATPase BRG1 (SMARCA4) or BRM (SMARCA2) (reviewed by [172]). While *Brm* is not required for early development [183], *Brg1*‐deficient blastocyst die before implantation and both the TE and the ICM are affected [174]. In the pluripotent state, SWI/SNF is present as a specific complex in ESCs, called esBAF [184, 185] and it was shown by deletion of esBAF components (*Brg1*, and *Baf250a* and *b*) that it is required for maintenance of the pluripotency network [184, 185, 186, 187, 188]. The core pluripotency TFs POU5F1 (OCT4) and NANOG and the LIF downstream effector STAT3 depend on BRG1 to bind to their targets. The functions of *Brg1* and pluripotency TFs are closely tied together as binding of the TFs and BRG1 continuously rely upon each other, so that BRG1 also requires the TFs for recruitment [170, 184, 189, 190, 191, 192]. The interdependency between POU5F1 and SWI/SNF seems to be remodeler specific as a screen for a link between binding of different chromatin remodeling complexes and POU5F1‐dependent ATAC sites showed that only BRG1 correlated [170]. Specificities between remodelers and TFs were also shown for ISWI and SWI/SNF chromatin remodelers that interact with different TFs for site‐specific recruitment to enhancers [193].

The role of SWI/SNF during the epiblast state and gastrulation has been less studied, as *Brg1* is crucial during pluripotency stages in embryos and ESCs [174, 185, 186] and conditional deletions are required to study subsequent roles. One study investigated the role of *Brg1* during ESC differentiation toward cardiac mesoderm using a conditional allele to delete *Brg1* starting from the epiblast stage [194]. This showed that *Brg1* is required for induction of general and cardiac‐specific mesoderm gene expression and therefore for differentiation of ESCs, most likely through its actions at enhancer regions. Also, here a TF seems to be involved in the functions of SWI/SNF. During differentiation toward cardiac mesoderm, MESP1, a crucial early TF for cardiac and other anterior mesoderm development [36, 195], is required for establishment of chromatin accessibility at its target sites [37]. In *Mesp1*‐deficient embryos, where cardiac progenitors are not generated, the chromatin landscape as measured by single‐cell ATAC‐seq is highly altered, suggesting that the TF MESP1 together with BRG1 controls accessibility of early mesodermal enhancers [41]. A recent study describes a similar function for the T‐box TF *Eomes* [42], that functions upstream of *Mesp1* as a general inducer of mesoderm and DE during gastrulation [34, 196, 197]. EOMES and BRG1 interact directly and rely on each other for binding to ME enhancers, where they establish accessible enhancer sites and activate the ME gene program during early gastrulation [42].

Overall, these results suggest that SWI/SNF chromatin remodeling relies on lineage specifying TFs like POU5F1 (pluripotency), EOMES (mesoderm and endoderm), MESP1 (anterior mesoderm), GATA6 (endoderm), and ASCL1 (neural specification) for recruitment to enhancers, where it establishes their accessibility [37, 39, 41, 42, 170, 171]. On the other hand, SWI/SNF seems to have a function in the maintenance of polycomb group (PcG) protein functions at bivalent promoters, that restrict expression of alternative or future lineage genes (Fig. 3). In the cardiac differentiation study, mesodermal genes were also upregulated after conditional deletion of *Brg1* [194]. These mesodermal genes are normally activated later during cardiac differentiation and their de‐repression is due to functions of *Brg1* in concert with PRC2 [194]. Normally SWI/SNF is thought to counteract the functions of PcG proteins [198, 199], and esBAF has been shown to remove PcG proteins from regulatory regions [191, 194, 200, 201, 202]. However, also here a mechanism was described where esBAF is required for continued function of PcG proteins at developmental gene promoters, as PcG protein binding is reduced upon loss of *Brg1* or *Baf250a* [191, 200, 203]. Furthermore, BRG1 was found to bind both active and inactive enhancers in many embryonic tissues also at later stages, suggesting that also there it impacts both active and repressed genes [204].

### The NuRD complex regulates transcriptional output for correct differentiation

In addition to SWI/SNF, the NuRD remodeling complex has important functions during pluripotency exit. The NuRD remodeling complex belongs to the chromodomain helicase DNA‐binding (CHD) remodeling complexes and contains in the blastocyst and the gastrulation stage embryo the ATPase subunit CHD4 [205, 206]. It is traditionally associated with a repressive role in transcription regulation, but has been shown to impact transcription in a more complex way (reviewed by [207]). A special feature of the NuRD complex is its histone deacetylase function in addition to the nucleosome remodeling function (either HDAC1 or HDAC2). Furthermore, it contains one of the two (methyl‐) CpG binding proteins MBD2 or MBD3 (reviewed by [208, 209]). Only *Mbd3* seems to play a role during early mouse development as *Mbd2*‐deficient mice are viable and fertile [210]. In the absence of *Mbd3*, the NuRD complex is disassembled [211]. *Mbd3*‐deficient embryos implant, but the epiblast does not develop further [177]. *Mbd3*‐deficient ESCs remained pluripotent in differentiation medium and did not commit to developmental fates beyond the epiblast state [211]. This phenotype is not simply caused by failed repression of pluripotency genes, but also by an overall failed regulation of correct levels of germ layer gene expression [211, 212, 213, 214]. To some degree, the NuRD complex seems to counteract the functions of BRG1 (SWI/SNF) in ESCs as genes are deregulated in an opposite manner upon removal of *Brg1* or *Mbd3* and both bind the same sites [215]. In line, several studies suggest that NuRD is required for the function of PRC2 by its deacetylase function. Deacetylation of H3K27ac by NuRD seems to be required for subsequent recruitment of PRC2 and methylation of H3K27 during silencing of active genes (e.g., pluripotency genes during differentiation) [206, 216].

Interestingly, NuRD is also localized to actively transcribed genes and has been shown to bind many active enhancers and promoters in ESCs [217]. Several studies suggest that the NuRD complex is required to fine‐tune transcription to intermediate levels to allow rapid inactivation of these genes during exit of the ESC state to differentiate toward specific lineages [214, 217, 218, 219].

The presence of the (methyl) ‐CpG binding protein MBD3 in the NuRD complex suggests that the complex could be recruited in a TF‐independent way. MBD3 does not bind methyl‐CpG [220, 221], but is suggested to be recruited to 5‐hydroxymethylcytosine, the product of TET enzymes [215]. The precise role of this recruitment has not been investigated. Evidence for TF‐mediated recruitment of the NuRD complex has been shown for ZIC2, which physically interacts with the NuRD complex and shares many binding sites where they are required for the pluripotent state [205]. *Zic2/3* have been described to be central in the TF network of primed pluripotency [48, 222, 223]. In addition, POU5F1 protein interaction studies have shown that, in addition to SWI/SNF, the NuRD complex also physically interacts with POU5F1 [224, 225].

In summary, both the SWI/SNF and the NuRD chromatin remodeling complexes are crucial during pluripotency and for subsequent lineage specification. Most conclusions are based on results from *in vitro* systems, as early embryonic lethal phenotypes make analysis in mouse embryos difficult. The SWI/SNF complex relies on lineage‐specific TFs to direct its action to stage and lineage‐specific enhancers and promoters where it is required for establishment and maintenance of the state by opening enhancers and promoters and keeping them accessible. NuRD is localized to active gene regulatory elements where it modulates activity to allow progression of development. Most likely this is necessary to allow the cells to stay in a transient state by making stabilization of the ESC state unlikely. Upon differentiation, SWI/SNF is recruited to alternative sites by the activation of lineage‐specific TFs. NuRD allows pluripotency genes to be deactivated by the recruitment of PRC2 and by counteracting the functions of SWI/SNF.

### Intracellular effectors of the NODAL signaling pathway interact with epigenetic factors to control gene expression

Extracellular signaling pathways are essential for pluripotency maintenance, pluripotency exit and for differentiation to cell lineages during gastrulation. The NODAL signaling pathway is crucial during formative pluripotency and for gastrulation initiation and ME cell lineage specification (see above and reviewed by [226, 227]). WNT signaling is required to maintain the naïve pluripotent state and also acts together with NODAL signaling during ME lineage specification (reviewed by [30, 228]). The intracellular effectors of these signaling pathways SMAD2/3 (NODAL signaling) and ß‐CATENIN (WNT signaling) have been shown to interact with epigenetic modifiers such as SWI/SNF components (e.g., BRG1) and p300/CBP in other model systems such as keratinocytes or cancer cells (reviewed by [229, 230] and, e.g., [231, 232, 233]). Using ESCs to study pluripotency and gastrulation, mostly the interactions of NODAL/SMAD2/3 with epigenetic factors have been studied (mostly in human ESCs) and little is known about the epigenetic effects of WNT3/ß‐CATENIN signaling.

SMAD2/3 has been shown to interact mainly with histone modifiers that are involved in active gene expression during pluripotency and lineage specification. SMAD2/3 interacts with MLL2, the COMPASS cofactor DPY30 and NANOG to control promoter H3K4me3 on pluripotency genes and on bivalent lineage genes in human ESCs [234]. The active form of SMAD2/3, pSMAD2/3 was further shown to interact with p300 to activate gene expression through H3K27 acetylation in P19 embryonal carcinoma cells, that can be differentiated toward germ layers [235, 236]. Finally, SMAD2/3 was shown to interact with the H3K27me3 histone demethylase KDM6B (JMJD3) to remove PRC2‐mediated H3K27me3 from developmental promoters to allow for their transcription for lineage differentiation [237, 238, 239]. However, not all SMAD2/3 interaction studies confirmed association with KDM6B [240, 241]. This regulation might lie upstream of WNT signaling, as WNT signaling alone was not able to induce removal of the PRC2 mark but could induce transcription in the absence of PRC2 in mESCs and human ESCs [237, 240, 242]. In summary, these studies show that the crucial NODAL signaling pathway has multiple impacts on the epigenetic regulation of pluripotency and lineage target genes. The site‐specific recruitment of SMAD2/3 proteins is most likely directed by lineage‐specific TFs [243], suggesting that SMAD2/3 could serve as a link between TFs and epigenetic modifiers.

### Pioneer TFs mediate H3K4me1 to initiate enhancer activation upstream of master TFs

**Fig. 4:**
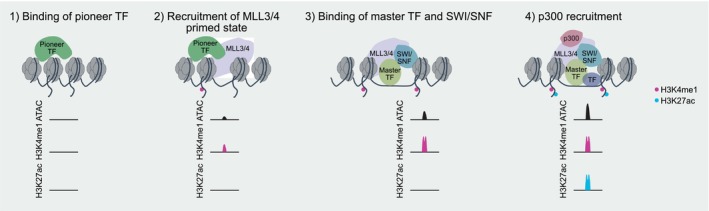
Sequence of events of enhancer establishment. Pioneer factors possess the ability to bind to closed, compacted chromatin (1). Pioneer transcription factors (TFs) recruit MLL3/4 and generate a primed state, which shows low chromatin accessibility and a single H3K4me1 peak (2). These primed sites are then bound by master TFs, which recruit chromatin remodeling complexes like SWI/SNF to establish an open, nucleosome‐depleted region, which is further characterized by a H3K4me1 double peak (3). Further cooperative transcription factor and p300 binding leads to full activation of the enhancer characterized by an additional increase in chromatin accessibility and gain in H3K27ac (double peak, 4).

What is the evidence for a role of TFs in the recruitment of other epigenetic modifiers? Interestingly, it was shown that FOXA1, which is a classical pioneer factor, recruits MLL3/4 to enhancers in breast cancer cells. This recruitment leads to a single H3K4me1 peak and MLL3/4 binding is strongly reduced at these enhancers in absence of *Foxa1* [244]. Most TFs only bind to a fraction of their consensus motif target site suggesting that chromatin state impacts TF binding. In line, many TFs are incapable of binding compacted chromatin. On the other hand, pioneer TFs bind to compacted chromatin establishing a primed epigenetic state (Fig. 4). This primed state is characterized by a single H3K4me1 peak and modestly accessible chromatin. Further activation requires cooperativity of master TFs, which then results in nucleosome‐depletion, increased H3K4me1 and chromatin accessibility as well as H3K27ac deposition by p300/CBP (reviewed by [72, 245] (Fig. 4)).

During early development, ZIC2‐binding sites in the ESC state also contain a large fraction overlapping with a single H3K4me1 peak [205]. Furthermore, the ME enhancers, which open during ESC differentiation, are premarked by a single H3K4me1 peak, which are then made accessible by the TF EOMES by recruitment of the SWI/SNF complex leading to a double H3K4me1 peak around the nucleosome‐depleted open region [42] (Fig. 4). However, it is not clear which factor establishes this single H3K4me1 peak at ME enhancers as ZIC2 has been proposed to show repressive functions on ME lineages [205, 222] and association with MLL3/4 remains to be shown. These data suggest that also during pluripotency transition and lineage specification pioneer TFs play a role and might recruit MLL3/4 to prime lineage‐specific enhancers. Master transcription factors like EOMES and MESP1 then fully activate these enhancers by establishing accessibility by interactions with SWI/SNIF [37, 41, 42]. Recruitment of further epigenetic modifiers then leads to full activation of enhancers (reviewed by [246]) (Fig. 4). At promoters, pluripotency TFs have been shown to also bind to developmental genes in ESCs and are there colocalized with PRC2 [120, 131, 247]. This could suggest that pluripotency TFs are involved in recruitment of PRCs to developmental target genes.

In conclusion, evidence points to the fact that stage and lineage‐specific TFs are responsible for recruitment of epigenetic modifiers. How pioneer TFs are recruited to the specific sites is currently investigated, as they also show lineage‐ or state‐specific binding patterns. Repressive histone marks or stage‐specific cofactors might be negatively and positively involved in pioneer TF recruitment [38, 48, 248] (reviewed by [72, 245]). Finally, this leaves the question open if TFs or an epigenetic modification are the first step in lineage decision.

## Passive competence at NE enhancers precedes active establishment of ME enhancers

During gastrulation in the mouse embryo, posteriorly restricted signaling dynamics of the NODAL, WNT, FGF, and BMP pathways lead to the induction of the PS and of mesoderm and DE lineages (Fig. 1). On the anterior side, the epiblast is shielded from these signals by secretion of extracellular antagonists from the anterior visceral endoderm (AVE) (Fig. 1). In this region in the absence of signaling pathways, the ectoderm germ layer is specified, which further develops into neuroectoderm and surface ectoderm. The development of neuroectoderm has been described as the default differentiation pathway from pluripotency as it develops in the absence of signals and as ESCs spontaneously differentiate along this path in the absence of other stimuli [249, 250, 251, 252, 253].

These fundamental differences in ME and NE lineage specification raises the question what the differences in epigenetic regulation at ME versus NE CREs are. The analysis of methylation and accessibility dynamics at lineage‐specific enhancers has shown that both differ between mesoderm/DE enhancers (ME enhancers) and NE enhancers [34, 84, 85]. Methylation at ME enhancers increases from 25% to 80% during pluripotency exit in embryos between E4.5 and E5.5 and is followed by a cell‐type‐dependent demethylation to 50% upon lineage specification. Surprisingly, NE enhancers are hypomethylated already at E4.5 and only become partially methylated upon ME specification (Fig. 2, light purple bars). It was shown that also chromatin accessibility at NE enhancers starts to be established by E4.5, whereas ME enhancers only become accessible later at initiation of gastrulation [84, 85] (Fig. 2, light purple bars). In line with accessibility of NE enhancers in the pluripotent state, another study showed that BRG1 was bound to CREs of neural genes in ESCs [184].

The analysis of enhancer histone mark dynamics in the mouse embryo during gastrulation showed that ME lineage enhancers were mostly quiescent (no histone marks) in the E6.5 epiblast and transitioned to primed (H3K4me1), bivalent (H3K4me1 and H3K27me3), or active (H3K4me1 and H3K27ac) between E6.5 and E7.5 [82, 83, 85]. About 40% of the NE enhancers already displayed active or poised histone marks at E6.5 correlating with early low methylation and chromatin accessibility. Quiescent NE enhancers transitioned to active/bivalent or primed states between E6.5 and E7.5 [83]. Furthermore, in human ESCs, enhancer bivalency is predominantly found at enhancers of anterior NE and less at earlier mesoderm and endoderm genes [119, 120]. This bias at the level of the enhancer landscape might contribute to the observed default differentiation‐path of pluripotent cells toward NE, in the absence of ME cell lineage promoting signals. In contrast to NE genes, enhancers of the early ME genes are generally chromatin inaccessible in pluripotent cells and only acquire enhancer bivalency during the earliest steps of ME lineage specification [37, 39, 42, 84, 85]. Although NE enhancer establishment precedes ME enhancer establishment, activation of ME gene promoters takes place by E7.0 while NE gene promoters are activated by E7.5. This shows that specification of ME and NE lineages happen at different developmental timepoints [83]. Therefore, whereas NE enhancers are demethylated, accessible and also to some degree marked by histone modifications before ME enhancers, the activation of promoters and gene expression takes place later.

The open and lowly methylated state of NE enhancers suggests that they are kept in a repressed state by other mechanisms during pluripotency and early gastrulation. The repressive mechanisms of NE genes during pluripotency have not been elucidated. An interesting candidate could be the repressive TF and epigenetic modifier REST, which has been shown to repress neural genes in non‐neural tissues (reviewed by [254, 255]). REST is present in ESCs and during differentiation of ESCs toward the neural lineage REST is degraded [256]. Repressive function of REST in ESCs are probably mediated by histone deactylases (HDACs) that are recruited by REST [256, 257]. Whether the polycomb group proteins are recruited by REST and involved in repression remains unclear [258, 259]. REST has been shown to interact with the SWI/SNF ATPase BRG1, which is required for REST function and recruitment [190, 193, 260, 261]. An important role for the other SWI/SNF ATPase *Brm* in NE repression during mesoderm differentiation of ESCs has been described. *Brm* restricts neuronal fate during early mesoderm specification by ensuring repression of neuronal genes through the TF REST [262]. In the embryo, *Brg1* seems to be able to compensate for loss of *Brm* [183, 262]. These data could fit with a role of REST in repression in ESCs, as NE enhancers are accessible and bound by SWI/SNF. However, evidence for a role of REST in the embryo during these early developmental steps beyond repression of neuronal genes in non‐neural cell types is so far lacking as deletion of REST does not lead to a gastrulation phenotype [263]. Therefore, the mechanisms that keep NE regulatory elements repressed during early development and the mechanism leading to activation of the NE program in the anterior epiblast remain to be fully described.

## Conclusions and future perspectives

The rapid evolvement of novel techniques over the last two decades such as CRISPR/Cas9 gene targeting and single‐cell multiome sequencing gave great insights into the functions of epigenetic modifiers in regulating cell identity and lineage‐specific gene expression. These studies demonstrated that the correct epigenetic regulation of both promoters and enhancers is required for appropriate activation of lineage‐specific gene expression. Promoters of developmental genes are chromatin accessible and DNA demethylated before their activation and are maintained in a repressed state by polycomb group proteins. Epigenetic changes at promoters of developmental genes are less dynamic and are not always correlating with lineage‐specific gene expression. However, a correct epigenetic state of promoters is required for induction of gastrulation gene programs as made clear by polycomb repressive complex 2 (PRC2) component mutants. Neuroectoderm enhancers are, like promoters, accessible, and demethylated before their activation, the repressive mechanisms are to date not clear. On the other hand, mesoderm and definitive endoderm enhancers are mostly in an inaccessible and inactive state before lineage specification and are activated in a lineage‐specific manner [84, 85] (Fig. 2, light purple bars). Their activation correlates with activation of gene expression. The primed enhancer state is established by pioneer transcription factors with the histone methyltransferases MLL3/4 and serves as a state that allows further activation by master transcription factors and other lineage‐specific transcription factors in concert with epigenetic modifiers (Fig. 3).

The epigenetic machinery seems to act in a highly redundant manner to ensure robust gene activation and repression to accomplish lineage specifications. This is true at the level of single modifications such as H3K4me3 placement, that is redundantly performed by several histone methyltransferases. It is also the case in a more complex way, as activation of gene expression can take place in absence of single epigenetic marks. The removal of H3K27ac at enhancers still allowed activation of genes as does the reduced demethylation in *Tet* triple KO embryos [102, 104, 168]. Moreover, the analysis of catalytic dead *Mll3/4* knock‐in mutants has shown that the recruiting functions of an epigenetic modifier can play a role in regulating gene expression even in the absence of the epigenetic mark (e.g., [125]). This indicates that robustness is not only ensured by the redundancy of factors to place a specific epigenetic mark, but also in the redundancy of epigenetic marks themselves in regulating gene expression. Consequently, single‐gene deletions of epigenetic modifiers in mouse embryos often do not reveal their functions as they are masked by redundant factors. Combined deletions of epigenetic modifiers establishing the same type of cis‐regulatory elements will give deeper insights into, for example, the requirement of bivalency. To investigate the effect of epigenetic modifications on cis‐regulatory elements of specific gene loci, catalytically dead Cas9 fused to epigenetic modifiers can be employed (reviewed by [264, 265, 266]). As the extraembryonic tissues substantially contribute to the signaling environment regulating gastrulation, newly developed *in vitro* models as ETX embryoids, which also consist of extraembryonic lineages, might provide more evidence for the sequence of events of gene activation during pluripotency exit and lineage specification in the gastrulating embryo.

Many studies establishing the roles of epigenetic modifiers and transcription factors during pluripotency and gastrulation have been performed in *in vitro* ESC systems. These model systems are great tools to study complex mechanisms as they are easier to manipulate than the embryo, especially in the case of mammalian embryos. However, in some cases results from embryos and ESC models do not align perfectly and are sometimes even contradictory (e.g., *Eed* (PRC2 complex)‐deficient embryos have a gastrulation phenotype with lack of mesoderm and neuroectoderm, while *Eed*‐deficient ESCs were shown to differentiate into all lineages [94, 133, 142], and, for example, bivalency is observed in cultures ESCs while in mouse embryos it is only detected by E6.5). This complication arises from the fact that ESCs are maintained in different culture conditions and that many different differentiation protocols exist, thus results may differ depending on the protocol and the time of analysis. In addition, the state of ESCs is only a transitional state in the embryo, whereas in culture, ESCs are maintained over long time periods in this state. The development of more complex *in vitro* systems such as gastruloids and ETX embryoids that resemble the *in vivo* complexity more reliably will probably help to resolve discrepancies. Still, care needs to be taken when interpreting results from *in vitro* systems.

Despite the crucial roles of epigenetic modifiers during gastrulation, it remains difficult to assign lineage‐specific functions. Evidence suggests that pioneer and master transcription factors provide specificity to epigenetic modifiers. It was shown for many lineage transcription factors that they establish accessibility at enhancers and in some cases that this is established by association of these transcription factors with the SWI/SNF remodeling complex (e.g., [170, 171]). For other epigenetic modifiers, the evidence is less extensive, but several studies also suggest interactions with transcription factors. This is also reflected in the phenotypes of transcription factor‐deficient mouse embryos that frequently show lineage‐specific defects (e.g., [195, 196, 197, 267]), while embryos deficient of epigenetic modifiers often display more general defects like developmental arrest (e.g., [94, 102]). However, the question whether the first step is defined by an epigenetic modification or a transcription factor is still not resolved, as also pioneer transcription factors show differential binding depending on the lineage.

## Conflict of interests

The authors declare no conflict of interest.

## Author contributions

KMS and SP wrote the Review.

## Peer review

The peer review history for this article is available at https://www.webofscience.com/api/gateway/wos/peer‐review/10.1111/febs.70024.
